# Shared Decision-Making on Life-Sustaining Treatment: A Survey of Current Barriers in Practice Among Clinicians Across China

**DOI:** 10.3390/healthcare13050547

**Published:** 2025-03-03

**Authors:** Yang Liang, Hua Zhang, Shu Li, Qingbian Ma

**Affiliations:** 1 Department of Emergency Medicine, Peking University Third Hospital, Beijing 100191, China; 2 Key Laboratory of Molecular Cardiovascular Sciences, Ministry of Education, Beijing 100191, China; 3 Clinical Epidemiology Research Center, Peking University Third Hospital, Beijing 100191, China

**Keywords:** decision-making conversation, life-sustaining treatment, barriers

## Abstract

**Background:** The perceptions of practicing physicians regarding the current situation and appropriateness of shared decision-making (SDM) in life-sustaining treatment (LST) are of essential importance. The objective of this study is to investigate the clinical practice patterns and barriers to this process in China. **Methods:** A cross-sectional survey of physicians in China was conducted to assess perceived practices of SDM in LST. The survey instrument was developed through the Delphi method. Descriptive analyses and an exploratory factor analysis were performed to explore the correlations of factors. **Results:** We polled 977 physicians, of whom 971 completed the survey and 834 (85.9%) have experience of decision-making conversations for LST and entered the final analysis. Physicians expressed that in the process of doctor–patient communication, over-expectations of disease prognosis (778 [93.3%]), lack of general medical knowledge (716 [85.9%]), and negative emotional status (574 [68.8%]) served as the main barriers to decision-making communication from the patient/family side, while under-expressed patient value (429 [51.4%]), limited communication due to work-load (359 [43%]), lack of communication skills (310 [37.2%]), and insufficient ability to cope with difficult cases (203 [24.3%]) as obstacles from the physician side. Only 185 (22.2%) physicians chose to disclose medical information according to literature evidence. The direct effects of physician service year and disclosure pattern on patient/family decision-making ability revealed statistically significant correlations (β_DMA<SY_ = 0.08, *p* = 0.035; β_DMA<DP_ = 0.40, *p* < 0.001), as did the effect of disclosure pattern on patient value expression (β_PV<DP_ = 0.88, *p* < 0.001). **Conclusions:** From physicians’ perspective, barriers of decision-making conversations correlated with multiple factors, including under-expressed patient value, insufficient patient/family decisional capacity or ability, and experience and communication patterns of physicians.

## 1. Introduction

Shared decision-making (SDM) is a process whereby decisions are shared by patients and physicians, informed by the best available evidence, and weighted in light of the patients’ characteristics and values [1,2]. Among the management categories, “invasive procedures” are most frequently cited as appropriate for SDM (71.5%) [3]. Emergency departments and intensive care units (ICUs) provide healthcare to severely ill patients and introduce the first opportunities for SDM on life-sustaining treatment (LST) to occur, with information shared between patients and clinicians across clinical disciplines [4,5]. Physicians should have a comprehensive understanding of patients’ conditions and needs to develop and adjust appropriate treatment plans in a timely manner during the treatment process. Similarly, patients must understand the purpose, risks, and effects of treatment to make informed decisions. Therefore, good decision-making communication between physicians and patients can improve treatment effectiveness and quality, thereby reducing the occurrence of medical errors [6].

Physicians have reported the use of SDM in situations that they deem appropriate but cite multiple barriers to its widespread use [7]. Emergency and ICU doctors typically experience particularly high levels of work-related stress and time restrictions. Their work is demanding due to the need to manage patients with diverse conditions and urgent medical needs while navigating the challenges of accurately assessing prognosis in life-threatening situations [5]. For patients who are seriously ill, “crisis communication” becomes challenging owing to less time for the development of patient–clinician relationships [8]. Previous studies have also highlighted that some physicians lack the necessary skills to perform SDM [9,10]. When patients are most vulnerable, the decision-making process is hardly consistent with what a reasonable patient would deem acceptable [11]. Many older individuals prefer to leave medical decisions to their providers or have difficulty communicating with surrogate decision-makers [5]. In particular, it is difficult to have decision-making conversations about unconscious patients or patients who require end of life care. The decision of life-sustaining therapies was based on respecting the patient’s presumed will, or in a futile outcome considered inappropriate by physicians. However, futility is neither objective nor universally recognized [12]. Additionally, patients or their surrogates often lack knowledge and deny the reality of the trajectory toward death [8]. Conclusions of studies on communication patterns remain controversial with no widely applied framework or protocol for serious illnesses. Thus far, many studies have explored patient perceptions and preferences regarding SDM [13,14,15]. However, few studies have investigated physicians’ perspectives regarding the communication they provide to their patients and related influential variables.

Moreover, considerable cultural differences exist between Asian and Western countries. Decision-making processes are often embedded in a framework of ethical and legal recommendations, which may vary between countries resulting in divergent management strategies. In Western countries, people emphasize individual autonomy and make personal decisions based on information from healthcare providers. Direct disclosure of diagnoses (e.g., terminal illness) is standard, and transparency is legally mandated. Although physicians typically support patients’ rights, many have reported a willingness to override the explicit directives of patients based on the requests of surrogates [16,17]. In Asia, people prioritize collectivist values, where legal systems may even accommodate family involvement [18]. Surrogates or proxies are almost equal to patient family members. Protective truth-telling is common; families may request withholding bad news to shield the patient. At present, people get to know about advance directives, but few people have made it written and signed as formal legal documents in China. Additionally, the legal effect of advance directives is only explicitly recognized through local legislation in Shenzhen, and there are no relevant provisions in national laws. The participation of patient family members in decision-making makes the dynamics of decision-making more complex.

This study investigates clinical practice patterns of how physicians recognize, initiate, and perform decision-making conversations regarding LST. It aims to further explore the systemic, cultural, and individual barriers, especially under Asian cultural and familial dynamics involving multiple variables that affect decision-making conversation from the perspective of physicians who treat patients in life-threatening situations in China.

## 2. Materials and Methods

We used an observational, descriptive, cross-sectional study design to survey physicians across China regarding their practices of SDM for LST. The method of cross-sectional study was chosen to efficiently collect data from a large sample at a single time point, promptly revealing prevalence, correlation, and patterns of phenomena.

### 2.1. Survey Instrument

A literature review was first conducted, and key factors during the SDM process for LST were identified and modified [6,8,19,20,21,22,23,24,25,26,27]. The initial draft of the survey instrument was created by authors with expertise in SDM. The questionnaire was developed and revised by a steering committee comprising emergency clinicians, academic researchers, and administrative authorities of healthcare and medical education who were experienced in LST and SDM.

The items in the questionnaire were further modified and finalized through a Delphi process (Supplementary File S1). The expert panel included emergency physicians, cariologists, respiratory intensivists, epidemiologists, medical science researchers, bioethicists, clinical medical ethicists, senior directors of the division of medical-legal affairs, representatives of educational administration, and presidents of hospitals, all with ≥15 years of experience. Individual items were scored using a 5-point Likert scale, with responses ranging from very insufficient (score of 1) to very good (score of 5). A coefficient of variance of >0.25 was considered as the rule-out criterion for the items. Kendall’s coefficient was calculated to evaluate expert consensus (<0.2, insufficient; 0.2–0.4, fair; 0.4–0.6, moderate; 0.6–0.8, good; and 0.8–1.0, excellent), where a value of 0.7 represented a valid cut-off criterion for the Delphi study or that no further insights nor improvement in answer quality were achieved from additional Delphi rounds.

The questionnaire was finalized through a two-round Delphi process, which reached a moderate consensus with no further insights (Supplementary File S2). We have tested internal validity, and the Cronbach’s alpha is 0.622 and it is acceptable. The questionnaire consisted of five domains with 33 questions (3–10 questions in each domain), including demographic variables, overall viewpoints on existing barriers, perceived treatment and prognosis, expressed decisional capacity and patient value, and physician communication competence (Table 1). The demographic variables included sex, age, level of hospital, specialty, educational background, work experience and seniority, work location, weekly working hours, experience with LST, and experience with decision-making communication training.

To ensure the validity and accuracy of the collected data, the investigators provided detailed instructions regarding the study objectives. To improve the quality of the answers, 10 emergency physicians were enrolled in a pilot study to evaluate the platform used and item comprehensibility. After preliminary data analysis, the final survey instrument underwent minor phrasing and formatting revisions before use.

### 2.2. Study Setting and Population

Our target research population is all clinicians who have participated in the decision-making conversation process for LST. Those who do not agree to participate in the study will be excluded. The doctor–patient communication for life support treatment of critically ill patients is usually carried out by physicians, and thus, nurses or other professionals are not included. Estimating the effect size of relevant studies, given the different issues, contexts, and subject populations, is difficult. Although the sample sizes of previous related studies have varied, a sample size of more than five times the number of questions is usually recommended to ensure more robust results [28]. We used the events per variable (EVP) method to estimate the sample size. We investigated 23 factors and calculated the EVP as 20, with a sample size of 460. A minimum sample size of 150 observations was defined after a comprehensive discussion with a clinical epidemiologist (ZH). Subsequently, the data collection period of the academic year was carefully chosen and justified; thus, the data for the main study were collected for one month.

All potential participants were recruited from a social network through convenience and snowball sampling due to limited resources. We start with a small number of initial participants (“seeds”) who meet the study criteria in our own hospital through WeChat and social media to send an online questionnaire link. We use multiple “seeds” from different departments (convenient sampling) to balance the sample distribution to minimize bias. Then, we conduct surveys with the initial participants and encourage them to refer additional participants who fit the study criteria in their network. Incentives (e.g., payment, vouchers) were not offered. New participants are asked to refer others, continuing the chain until data saturation is reached (no new participant emerges). Participants were deemed non-respondents if they declined to participate in the survey. To enable nationally representative results, data were collected using an online questionnaire tool, WJX (https://www.wjx.cn/vj/YCJ1pwU.aspx (accessed on 30 April 2022)). Confidentiality measures were taken to protect participants. The platform allows participants to fill out the questionnaire without registering or logging in. The questionnaire separates sensitive and non-sensitive questions and did not collect direct identification information. Moreover, potential identification fields (e.g., timestamps and IP address) were removed when exporting data. Access to raw data is restricted to authorized personnel, and raw data will be promptly deleted upon research completion, with only anonymized results retained. Participants were fully informed prior to participation that the questionnaire is anonymous, and they were made aware of the purpose of the data and confidentiality measures. Neither missing nor duplicate values needed to be considered because the survey tool automatically verified that all critical questions had to be completed before submission and could not be submitted twice.

### 2.3. Statistical Analysis

For all statistical tests, *p*-values were derived from two-tailed tests, and the results were considered statistically significant when *p* < 0.05. Data analysis was conducted using IMB SPSS Statistics for Windows (version 22.0; SPSS Science Inc., Chicago, IL, USA) and the R programming language 4.3.2 for Windows (R Core Team [2014], R: A language and environment for statistical computing. R Foundation for Statistical Computing, Vienna, Austria, http://www.R-project.org.) through the graphical user interface RStudio (RStudio Team [2020]. RStudio: Integrated Development for R. RStudio, PBC, Boston, the US. http://www.rstudio.com/).

Statistical analyses were performed between May 2022 and January 2024. Data were collected using WJX, and the source data were exported as EXCEL files from the website for further analysis. Individual survey responses were used as the units of analysis. For the descriptive statistics, mean and standard deviations were reported for normally distributed continuous variables, while median and interquartile ranges (IQRs) were reported for nonnormally distributed continuous variables. Frequency and proportions were reported for categorical variables.

There are no recognized scales for SDM in LST exist; therefore, an exploratory factor analysis was used. We aimed to explore the correlations of factors in the models, which would further allow us to assess interaction between those factors and their impact on the existing barriers of decision-making conversation. The factors with eigenvalues > 1 were screened and rotated using an orthogonal rotation method, and the analysis was performed using the *psych* package (version 2.4.12). Based on the results of the exploratory factor analysis and clinical experience, a structural equation model (SEM) was established. All SEM analyses were performed using the *lavaan* package (version 0.6-19), with a weighted least squares means and variance (WLSMV) estimation method. The Tucker–Lewis index (TLI), standardized root mean square residual (SRMR), root mean square error of approximation (RMSEA), normed fit index (NFI), ratio chi-square and degrees of freedom (χ^2^/*df*), and comparative fit index (CFI) were used as goodness-of-fit indices. The fit of the models was considered good if χ^2^/*df* was <5; SRMR and RMSEA values were <0.08; and CFI, NFI, and TLI values were >0.95. The ω, based on the polychoric correlation matrices, was calculated for first-order factors. For second-order factors, the variance of the first-order factors explained by the second-order factor (ω*L2*), the proportion of variance explained by the second-order factor after partialling the uniqueness of the first-order factor (ω*partial L1*), and the proportion of the second-order factor explaining the total score (ω*L1*) were calculated. All internal consistency estimates were obtained using the *semTools* package (version 0.5-6), and the structural model was analyzed through the SEM technique using the *lavaan* package, which was implemented through a two-step approach. Confidence intervals (95%) were provided for all paths, and the same criteria that were established in the evaluation of the measurement models were used to assess the goodness-of-fit of the latent variable structural model.

Here are some definitions of key concepts in equations. Patient value includes core principles (e.g., autonomy, family well-being, religious beliefs, quality of life) of patients and their specific choices aligned with those values (e.g., preferring palliative care over aggressive treatment). Decisional capacity/ability consists of three elements: (1) the possession of a set of values and goals necessary for evaluating different options; (2) the ability to communicate and understand information; and (3) the ability to reason and to deliberate about one’s choices [29]. Disclosure pattern means the way and types of medical information presented by physicians during doctor–patient conversation [30]. Physician experience means the work experience of doctors on the efficiency of patient communication, or challenges in SDM during the postgraduate delivery of healthcare directly to patients.

### 2.4. Ethical Approval

This study was approved by the Institutional Review Board of Peking University Third Hospital (approval number: IRB-2021-493). All participants were informed of the survey’s purpose, and electronic informed consent was obtained before answering the questions. Once the study was accepted by the participants, they were allowed to proceed with the questionnaire. 

## 3. Results

### 3.1. Participant Demographic Characteristics

**Figure 1:**
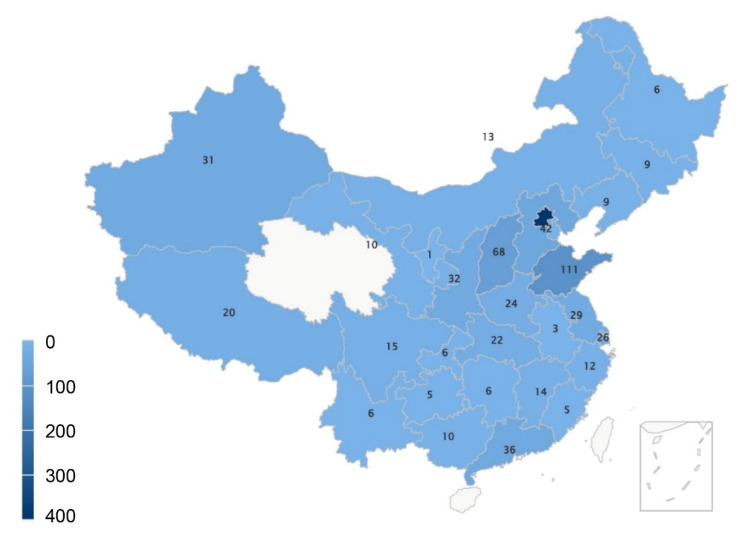
Geographic distribution of the responding physicians.

The survey data were collected between 17 March 2022 and 30 April 2022, and complete surveys were submitted by 971 of the 977 physicians who consented to participate. The response rate was 99.4%. The enrollees of the survey were from 29 of the 34 administrative regions of China, and the geographic distribution of the responding physicians is shown in Figure 1. A total of 834 (85.9%) individuals who have participated in the decision-making conversation process for LST entered the final analysis. The median age of the physicians was 35 (IQR 30,42) years, and 481 were women (57.7%). The median length of service in healthcare facilities was 10 (IQR 5,17) years, and 696 (83.5%) respondents worked in tertiary hospitals. A total of 228 senior physicians (27.3%) were included, and 364 physicians (43.6%) claimed that they “often” performed cardiopulmonary resuscitation (CPR) (Table 1).

### 3.2. Main Barriers of Decision-Making Conversation

Physicians expressed that in the process of doctor–patient communication, the patient/family’s over-expectations of disease prognosis (778 [93.3%]), lack of general medical knowledge (716 [85.9%]), and negative emotional status (574 [68.8%]) served as the main barriers, while under-expressed patient value (429 [51.4%]), limited communication due to workload (359 [43%]), lack of communication skills (310 [37.2%]), and insufficient ability to cope with difficult cases (203 [24.3%]) were obstacles from the physician side (Table 2).

### 3.3. Decision-Making Model and Patient Values Expression

In total, 403 (48.3%) physicians described that they adopted an SDM process during decision-making conversations of LST, 60 (7.2%) valued patient-centered preference, 328 (39.3%) chose to follow family choice, and 43 (5.2%) dominated the whole process in paternalism mode. Furthermore, 79 (9.5%) physicians considered critically ill patients as fully capable, 255 (30.6%) as mostly capable, and 328 (39.3%) as partly capable, while only 172 (20.6%) considered them as having no decision-making capacity. Under these circumstances, 774 (92.8%) participants tended to accept opinions from the patients’ families, and 60 (7.2%) directly inquired with the patients. Among the study cohort, 87 (10.4%), 187 (22.4%), 289 (34.7%), and 271 (32.5%) physicians always, sometimes, occasionally, and never asked about patients’ advanced directives (AD) during conversation, respectively. Moreover, 347 (41.6%), 255 (30.6%), 170 (20.4%), and 62 (7.4%) physicians always, sometimes, occasionally, and never asked patients’ families about their previously expressed LST preferences, respectively.

### 3.4. Decisional Abilities of Patients/Families

A total of 96 (11.5%) physicians considered patients/families to have insufficient or extremely insufficient abilities to make rational medical decisions in the context of LST. Additionally, 107 (12.8%) physicians considered patients/family to have insufficient or extremely insufficient understandings of the necessity and urgency of LST. A total of 207 (24.8%) physicians considered patients/families to have insufficient or extremely insufficient comprehensions of the risk and prognosis of patients receiving LST (Table 3). Additionally, 774 (92.8%) physicians considered the decisions of the patient/family usually or sometimes concordant with physician judgment. Lastly, 665 (79.7%) physicians thought that patients/families usually or sometimes adhered to their decisions (Table 4).

### 3.5. Features of Communication Patterns

#### 3.5.1. Prognosis Evaluation and Disclosure

The factors that physicians considered appropriate to evaluate whether patients could benefit from receiving LST included disease prognosis (793 [94.7%]), comorbidity and living status (732 [87.5%]), patient age (689 [82.3%]), patient values (658 [78.6%]), and treatment expenses (373 [44.6%]). In addition, 558 (66.9%), 185 (22.2%), 74 (8.9%), and 17 (2.0%) physicians usually, sometimes, rarely, and never reviewed patients’ comorbidities and daily living statuses, respectively, during discussions with patients/families. Of the study cohort, 815 (96.7%) physicians usually or sometimes explained the main problems and general prognoses of diseases. A total of 743 (89.0%) physicians usually or sometimes mentioned alternatives to LST, such as epinephrine and oxygen masks. Of the study population, 779 (93.4%) physicians usually or sometimes mentioned the consequences of forgoing LST, such as possible death of the patient (Table 5).

#### 3.5.2. Objectiveness of Risk–Benefit Interpretations

If patients/family members inquired about the success rate of CPR, 649 (77.8%) physicians answered that it was difficult to predict precisely for each individual, while 185 (22.2%) chose to estimate the success rate according to literature evidence. Furthermore, when physicians were asked about the exact rate of primary success of return of spontaneous circulation (ROSC) for in-hospital cardiac arrest (IHCA), only 301 (31.1%) provided the correct answer, while 306 (31.6%) underestimated, 217 (22.4%) overestimated, and 143 (14.8%) rated “I have no idea” as their response. When physicians were asked about the exact rate of bone fraction caused by chest compressions, 411 (42.5%) underestimated the incidence, and 120 (12.4%) responded “I have no idea”. Furthermore, 550 (66.0%) physicians occasionally or never used tools (e.g., web videos, pictures) to communicate about LST.

When a patient is facing life-threatening needs for LST but their family members are not present/have not consented, 728 (87.3%) physicians would choose to immediately and actively take necessary measures to contact the family members and obtain their consent before deciding on the next step of treatment, while 101 (12.1%) selected to follow an active code or “slow code” based on their evaluation of the prognosis and ability to contact family members during that time.

#### 3.5.3. Time and Opportunity of the Conversation

A total of 343 (41.1%) physicians claimed that they finished conversations within 5 min, while 206 (24.7%) took 5 to 15 min. Of the physicians, 711 (85.3%) thought that they should talk about CPR when any potential for its use was present, whereas 121 (14.5%) thought that CPR should be appropriately discussed when a patient was deteriorating.

### 3.6. Exploratory Factor Analysis

**Figure 2:**
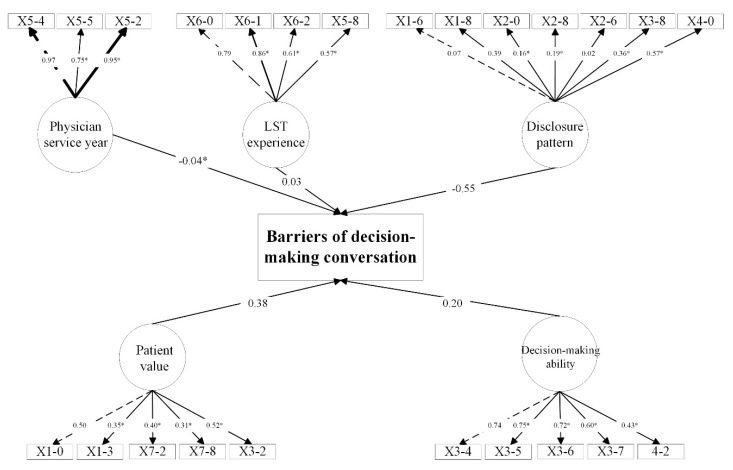
The variable-structure model.

An exploratory factor analysis was performed, and relevant factors were identified (Supplementary File S3 presents all regression paths). SEM was established based on the exploratory factor analysis results. The CFI was 0.864, and the TLI was 0.843. The variable-structure model presented a good fit to the data, and the direct effects of most factors were statistically significant (Figure 2). The direct effects of physician service year and disclosure pattern on patient/family decision-making ability revealed statistically significant correlations (β_DMA<SY_ = 0.08, *p* = 0.035; β_DMA<DP_ = 0.40, *p* < 0.001), as did the effect of disclosure pattern on patient value expression (β_PV<DP_ = 0.88, *p* < 0.001). While disclosure pattern, patient value, and decision-making ability did not present a statistically significant effect on barriers of decision-making conversation, the effect size was not negligible (β_BDPC<DP_ = −0.55, *p* = 0.166; β_BDPC<PV_ = 0.38, *p* = 0.197; β_BDPC<DMA_ = 0.20, *p* = 0.829).

Structural equation model and standardized structural coefficients. List of factors: X5-4: year of service; X5-5: title; X5-2: physician age; X6-0: code leader; X6-1: familiar with LST; X6-2L: frequency of code activation in past month; X5-8: field of expertise; X1-6: conversation based on physician habit or patient’s condition; X1-8: review comorbidity and daily living status; X2-0: explain the main issue and general prognosis; X2-6: mention the consequences of forgoing LST; X2-8: mention alternatives to LST; X3-8: explain actively; X4-0: use tool; X1-0: acquirement of advance directive; X1-3: patient decisional capacity; X3-2: ask for patient value; X7-2: consent to patient; X7-8: respect patient value; X3-4: understand the necessity and urgency of LST; X3-5: comprehension of risk and prognosis of patients receiving LST; X3-6: patient/family decision-making ability; X3-7: stick to decision; X4-2: patient/family decision concordance with physician. *: *p*< 0.05.

## 4. Discussion

The formal study of SDM is a relatively new trend in emergency medicine [3,4,6,14,31], and previous studies have suggested that SDM is often appropriate in medical environments. Despite the reported benefits of SDM, the results of our study reveal that challenges exist when integrating SDM into actual clinical practice. This study contributes information of decision-making conversations on LST from the perspective of physicians, and it is the first study to apply an exploratory factor analysis. It provides actionable insights to harmonize doctor–patient conversation with patient-centered care imperatives, cultural values, and legal reforms. Barriers of decision-making conversations correlated with multiple factors, including under-expressed patient value, limited patient/family decisional capacity or ability, and experience and communication patterns of physicians. Our findings show that patient value under-expression and insufficient patient/family decision-making ability might contribute to barriers of decision-making conversation from the perspective of physicians. The physician disclosure pattern attenuates this impact and works as a protective factor. In addition, higher titles for physicians and greater numbers of service years lend to less obstacles.

### 4.1. Physician Population

Nearly 1000 physicians responded to our survey, half of whom were women, and the distribution of seniority was relatively balanced. The vast majority of physicians from administrative regions across China participated in the questionnaire; however, most of those who responded were concentrated in tertiary hospitals in the north, which may be related to the distribution and collection methods of the questionnaire. The distribution of various specialties mainly focused on emergency medicine and internal medicine doctors, which relates to the attention and natural distribution of topics in different specialties but also includes a considerable proportion of surgeons and critical care physicians. Most of the physicians often performed CPR and were familiar with the discussion topics. More than half of the physicians worked > 50 h per week, indicating that their clinical work load was relatively heavy. Approximately three-quarters of the physicians had received training in decision-making communication.

### 4.2. Patient Value Inadequately Expressed

To align with patients’ life desires and care, physicians must explore aspects most significant to the patients and provide appropriate recommendations and interventions based on their preferences [27,29]. In Western countries, patient autonomy is defined as respecting patients’ rights to self-determination and is given prime importance in medical decision-making [32]. AD facilitates people’s self-determination and a national survey on association between AD and quality of end-of-life care in the United States showed that 70.8% had an AD of the 1587 people who died [33]. Recent studies showed a lower prevalence of the use of AD (10–16%) in different countries [29]. The discussion of preferences and priorities depends on each patient’s trajectory, decisional capacity, and urgency of decisions. Some patients lose their decision-making capacities due to underlying or existing diseases, and some conscious patients find participation in decision-making communication difficult because of factors such as pain, fear, and shock. Respect for autonomy may require overriding a patient’s stated treatment preference when capacity determination is equivocal [34]. For critically ill patients, the target audience for communication was a proxy even in the United States [35]. The preparation of family, friends, surrogates, caregivers, healthcare proxies, and substitute/medical decision-makers is also important. Furthermore, communicating with patients about the code status may cause significant mental stress. In our study, almost 80% of the physicians considered their critically ill patients to be at least partially capable of decision-making. However, 92.8% of the physicians in our study had decision-making conversation for LST with patients’ family members, while only 7.2% of physicians had decision-making conversations with the patients themselves. Notably, in a study by Anselm et al., which interviewed nurses and physicians, similar family-related barriers were identified in Canada, including shielding of patients from discussions, difficulty assigning a decision-maker, impact of different cultural and religious values, and understanding the capacities of family members [36]. Another study from Hong Kong with a predominantly Chinese population showed similar family-related issues in code status discussions, including decisions regarding end-of-life care, withdrawal of support being made by family members rather than the patient, and protecting patients from disclosing grave disease prognoses [37]. Families are often overwhelmingly involved in LST discussions in China because it is a relatively family-centered society, and individuals are viewed primarily as a part of a family. Even if patients initially participate in the decision-making process and express their wishes, in the event of declining health status, the patient’s wishes may not be implemented due to inconsistencies with the family members’ opinions or difficulties in accepting sudden changes. Meanwhile, only about one-third of the physicians mentioned patients’ advanced directives in our study. Fortunately, approximately 70% of physicians inquired about patient preferences through family members and asked questions regarding whether patients had discussed or expressed the idea of accepting LST at the end of their lives. The proportion of successful expressions of overall patient willingness is not optimal and is constrained by multiple factors, including patients’ sociocultural background. Therefore, we advise caution in interpreting the raw percentages of the responses and suggest that readers focus on the relative appropriateness of the different clinical scenarios when it is impossible or considerably difficult to ask the patient about their wishes. We found that better disclosure patterns potentially have positive impact on patient value expression. Furthermore, it is critical to develop national guidelines on LST decision-making, integrating lessons from regional pilots. The designation of surrogate and advance directives should be proposed during conversations to empower patients and emphasize their autonomy.

### 4.3. Gap Between Patient/Family Comprehension and Disease Complexity

Providers often overestimate patient understanding [5,38]. In our study, physicians believed that the decision-making ability of patients/family members was insufficient, and approximately 20% held a wavering attitude toward decisions. The appropriateness and desirability of LST may differ between patients, families, and providers. Patients/family members often have an insufficient understanding of the necessity and urgency of LST [36], which may relate to their understanding of the medical condition and prognosis. For critically ill patients, especially those with multiple organ dysfunction and shock, the manifestation of the condition is intuitive, with a certain environmental foundation for discussing LST. However, patients with clear consciousness, stable vital signs, and only a potential risk of cardiac arrest, such as those with arrhythmia or acute myocardial infarction, usually underestimate risk and may not fully understand the necessity of LST. However, when offered a choice, many patients with grave prognoses prefer more aggressive care than what is necessary [26]. Patients/families may have cognitive biases toward LST, with confusion between chest compressions and defibrillation, overestimation of the success rate of CPR, and underestimation of the risk of sternum/rib fractures. Patients in previous studies have cited television as a large contributor to the belief that the rates of survival after CPR vary between 19% and 75%, whereas actual rates of survival after CPR range from an average of 12% for out-of-hospital cardiac arrests to 24–40% for in-hospital arrests [39]. Overall, physicians must aim to strengthen the disclosure of facts and provide adequate education to patients/family members, enabling them to understand the principles, processes, application scenarios, necessity, success rates, potential trauma, risks, and alternative solutions of LST and filling any cognitive gaps.

### 4.4. Physician Efforts and Discrepancies

Our study also found a direct positive effect of physician experience and disclosure patterns on patient/family decision-making ability. Physicians should endeavor to communicate more effectively with patients and their families regarding their treatment preferences. Evaluating patient prognosis is a complex process that depends on comorbidities, daily living status, expected functional outcome, burden of treatment, and medical intervention preference based on patient’s and family’s values [40,41,42,43,44]. While most physicians discussed disease prognosis, 10% of the physicians did not consider patients’ medical history during communication. Furthermore, only 22.2% of the physicians quoted the success rate of CPR based on evidence-based medicine. However, when asked about the success rate of CPR and incidence of fractures, a considerable number of physicians were unaware of the correct answers. The corresponding knowledge and evidence-based thinking must be improved to allow clinicians to provide objective information to their patients. Regarding conversation content, >90% of physicians chose to mention the consequences of forgoing LST, and nearly 90% of physicians mentioned alternative solutions. An alternative to LST is to not perform this treatment, and other options are still available, including adrenaline and oxygen masks. This communication can reduce misunderstandings equating “not undergoing invasive rescue”, with “giving up treatment”, or “not receiving treatment at all”. Under the pressure of moral guilt or public opinion, families may request that physicians perform every possible life-saving procedure. Only 34.1% of the physicians in our study used patient decision tools during conversations. Education and support in the decision-making process can be provided using patient decision tool involvement [42,43,44,45,46]. Decision tools provide information on the risks and benefits of available treatments and elicit patient values regarding these choices. Transforming content directly from the evidence base into easily understood formats may facilitate patient–provider communication. A survey of nephrologists and internal medicine trainees from Korea showed that only 12.3% of respondents had received education on SDM as part of their training. The main obstacles to appropriate SDM were identified as lack of time (46.0%), educational materials and tools (29.4%), and education on SDM (24.3%) [47]. Implementing training programs for physicians on evidence-based and culturally sensitive communication is crucial in the future.

Notably, 85% of physicians tried to persuade patients/family members when they believed that their decisions were inconsistent with their expectations to prevent potential misunderstandings. Furthermore, 12.1% of the physicians chose to run a “slow code” when they thought that CPR was not beneficial. While some clinicians believe that this practice is harmful and deceptive, disregarding patient and surrogate autonomy, others argue that a slow code is a non-maleficent and beneficial act toward very ill patients with poor chances survival [48]. A survey by Pisctello et al. showed that a narrow majority of respondents (52%) reported that slow codes were ethical if they were medically futile. Meanwhile, 19% and 28% believed that no code and a full guideline-consistent code should be used, respectively. Most respondents reported moral distress when required to run (75%), perform chest compressions (80%), or witness (78%) a cardiac resuscitation attempt that they believed to be medically futile [49].

## 5. Limitations

A critical weakness of this study is its non-longitudinal approach, which allows the study of correlations but does not necessarily lead to causal interpretations. The lack of certainty regarding the stability of its measures decreases the internal validity of our study. Second, we surveyed a nonrandom sample of physicians during the COVID-19 pandemic, which may influence our conclusions. Physicians who responded to the recruitment notice were self-selected and may differ systematically from the overall physician population. Furthermore, physicians in tertiary hospitals in North China were overrepresented in our study cohort and may have different perspectives on SDM than physicians who practice in community settings or rural areas, potentially introducing bias. Each region’s sample was insufficient and did not allow for multigroup analysis, comparison between different regions, or external validation of these results. However, we recruited 168 community physicians to complete the survey. Additionally, the study distribution reflects the attention of professionals who frequently perform CPR and ensures a collection of opinions from this population. As in any survey study, the potential for social desirability bias is present. Third, our survey study was only able to measure what physicians reported but not what they performed. Moreover, our sample represents a relatively small number of physicians, although this is the most effective way to obtain input from across countries. Fourth, our survey sought to elicit opinions from physicians but did not elicit opinions from nurses, patients, educators, administrators, or policymakers. Finally, no known scales for SDM in CPR exist; therefore, an exploratory factor analysis was used. Notably, the correlation coefficients of the indicators were not ideal.

## 6. Conclusions

Based on the results of our cross-sectional study, the practice of SDM among physicians surveyed on LST does not appear to be ideal. Overall, SDM was widely endorsed by physicians in our cohort; however, further work is needed to elucidate how to improve clinical practice for decision-making support and conversations. From the physicians’ perspective, the patients’ values were insufficiently expressed. Physicians should provide evidence-based information and a comprehensive explanation of both past and current medical conditions to foster better mutual understanding. Education and support in the decision-making process can be provided using patient decision tool involvement. To bridge this gap, investigating patient and family perspectives to align physician practices with societal expectations and implement training programs for physicians on culturally sensitive communication are both directions of efforts in the future. At policy level, it is critical to develop national guidelines on LST decision-making, integrating lessons from regional pilots.

## Figures and Tables

**Table 1:** Demographic characteristics of the included physicians.

Characteristics	N of Physicians (%)
Sex, female	481 (57.7)
Education background, doctor’s degree in medicine	557 (66.8)
Years of medical practice	
<10 years	453 (54.3)
10–20 years	259 (31.1)
>20 years	122 (14.6)
Title	
Senior	228 (27.3)
Median	325 (39.0)
Junior	281 (33.7)
Hospital level, Tertiary hospital	696 (83.5)
Specialty	
Emergency medicine	264 (31.7)
Internal medicine	198 (23.7)
Surgery	101 (12.1)
Intensive medicine	90 (10.8)
Cardiology	77 (9.2)
Work time per week	
40~50 h	336 (40.3)
50~60 h	286 (34.3)
>60 h	212 (25.4)
Performing CPR	
Often	364 (43.6)
Sometimes	272 (32.6)
Occasionally	184 (22.1)
Never	14 (1.7)
Performing CPR during past month	
≥5 times	96 (11.5)
2~4 times	204 (24.5)
≤1 time	534 (24.8)
Previous doctor–patient communication training	622 (74.6)

**Table 2:** Main barriers to doctor–patient LST conversations identified in the study cohort.

Main Barrier to Doctor–Patient Conversations	N of Physician (%)
Patient/family member over-expectations of disease prognosis	778 (93.3)
Patient/family member lack of medical knowledge	716 (85.9)
Patient/family member’s negative emotional status	574 (68.8)
Neglected patient autonomy	429 (51.4)
Lack of time due to physician workload	359 (43.0)
Lack of communication skills of healthcare professionals	310 (37.2)
Insufficient physician ability to cope with difficult situations	203 (24.3)

**Table 3:** Decisional ability of patients/families.

Decision Category	Excellent	Good	Moderate	Insufficient	Extremely Insufficient
Decisional ability, *n* (%)	73 (8.8)	303 (36.3)	362 (43.4)	86 (10.3%)	10 (1.2%)
Understanding of the necessity and urgency of LST, *n* (%)	82 (9.8)	279 (33.5)	366 (43.9%)	91 (10.9%)	16 (1.9%)
Comprehension of risk and prognosis of patients receive LST, *n* (%)	45 (5.4)	154 (18.5)	428 (51.3%)	173 (20.7%)	34 (4.1%)

**Table 4:** Decisional appropriateness of patients/surrogates.

	Usually	Sometimes	Occasionally	Rarely
Concordant with physician judgment, *n* (%)	577 (69.2)	197 (23.6)	52 (6.2)	8 (1.0)
Stick to decisions, *n* (%)	399 (47.8)	266 (31.9%)	158 (18.9%)	11 (1.3%)

**Table 5:** Prognosis evaluation and disclosure to the patient.

Decision Category	Usually	Sometimes	Occasionally	Never
Review the patient’s comorbidities and daily living status, *n* (%)	558 (66.9)	185 (22.2)	74 (8.9)	17 (2.0)
Explain the main issue and general prognosis, *n* (%)	758 (90.9)	57 (5.8)	16 (1.9)	3 (0.4)
Used tools (e.g., web videos, pictures), *n* (%)	88 (10.6)	196 (23.5)	246 (29.5)	304 (36.5)
Mentioned alternatives to LST, *n* (%)	580 (69.5)	163 (19.5)	74 (8.9)	17 (2.0)
Mentioned the consequences of forgoing LST, *n* (%)	640 (76.7)	139 (16.7)	38 (4.6)	17 (2.0)

## Data Availability

The datasets used and/or analyzed during the current study are available from the corresponding author on reasonable request.

## Supplementary Materials

The following supporting information can be downloaded at: https://www.mdpi.com/article/10.3390/healthcare13050547/s1, File S1: Delphi study; File S2: Questionnaire; File S3: Exploratory factor analysis and structural equation model.

## Abbreviations

AD	advanced directives
CFI	comparative fit index
CPR	cardiopulmonary resuscitation
GOC	goals-of-care
ICU	intensive care unit
IHCA	in-hospital cardiac arrest
IQR	interquartile range
LST	life-sustaining treatment
NFI	normed fit index
RMSEA	root mean square error of approximation
ROSC	return of spontaneous circulation
SDM	shared decision-making
SEM	structural equation model
SRMR	SRMR, standardized root mean square residual
TLI	Tucker–Lewis index
WLSMV	weighted least squares means and variance
