# Supplementary material for: Shared Decision-Making on Life-Sustaining Treatment: A Survey of Current Barriers in Practice Among Clinicians Across China
Source: Healthcare (Basel). 2025 Mar 3;13(5):547. doi: 10.3390/healthcare13050547 (PMC11898668; doi:10.3390/healthcare13050547)
Supplement: Supplementary file 1 [file healthcare-13-00547-s001.zip › supplementary file S1 Delphi study.pdf]

S1. Method

This study used the Delphi method to form a questionnaire on the barriers in practice of life-sustaining treatment among physicians through two rounds of expert surveys.

S1.1 Implementation steps of Delphi method

S1.1.1 Research objective

Design a questionnaire for physicians' life-sustaining treatment practice.

S1.1.2 Establish a research team

The main task of the research project team is to construct a survey questionnaire based on the current situation of clinical work; identify Delphi expert panel; Distribute and collect questionnaires; Organize and provide feedback on the questionnaire; Report the results.

S1.1.3 Identify Delphi experts

Inclusion criteria: (1) frontline faculties, researchers, and policy-makers through the China Consortium of Elite Teaching Hospitals; (2) including clinicians with expertise in a variety of disciplines related to SDM of life-sustaining treatment; (3) deeply involved in clinical, research, teaching or administrative work related to SDM with more than 10 years' experience.

S1.1.4 Questionnaire Design

The questionnaire includes research introduction, informed consent, instructions, demographics, and confidentiality instructions. The importance of indicators is classified into five levels based on Likert, with responses ranging from very insufficient (score of 1) to very good (score of 5).

Collect expert opinions to form the next round of questionnaires, and provide qualitative feedback on the results of the previous round of questionnaires. Follow the above process for multiple rounds of consultation until the expert

opinions are unanimous.

S1.2 Data Management and Statistical Analysis Methods

Use Microsoft Excel 2016 for dual input of questionnaire content. SPSS 22.0 software was used for statistical analysis. The expert positivity coefficient is represented by the questionnaire response rate. Experts' familiarity with the survey is divided into "very familiar, familiar, average, unfamiliar, very unfamiliar", with scores of 1, 0.8, 0.6, 0.4, and 0.2. The evaluation criteria for indicators are assigned values as shown in Table 1. The authority of an expert is the arithmetic mean of familiarity and the coefficient of judgment basis. The degree of coordination of expert opinions is represented by the coefficient of variation and Kendall coefficient. A coefficient of variance of >0.25 was considered as the rule-out criterion for the items. The significance level of Kendall coefficient is set to 0.05. When the coordination coefficient exceeds 0.7 or the coordination coefficient of the second-round questionnaire is greater than that of the first round, the questionnaire will be terminated.

Table S1: Expert Judgement for Evaluating Indicators and Assigned Values

Judgment criteria	Level		
	Major	Medium	Minor
Practical experience	0.5	0.4	0.3
Theoretical analysis	0.3	0.2	0.1
Literature	0.1	0.1	0.05
Intuition	0.1	0.1	0.05

S2 Results

S2.1 Duration of study

This study conducted two rounds of questionnaire surveys, lasting for one month.

S2.2 Expert panel

A total of 8 experts participated. Among them, there are 2 males and 6 females with a median age of 46 years old. Experts are mainly those over 40 years old, with senior professional titles and more than 15 years of work experience in their respective fields, as shown in Table 2. Two rounds of questionnaires with a 100% response rate, indicating a high level of enthusiasm from experts. The authority scores of experts are above 0.8. Indicating a high level of expert authority and reliable results.

Table S2 Demographics of Expert panel

	Number of people (%)
Gender (female)	6(75.0%)
Age (>40 years old)	7(87.5%)
Title (Senior)	6(75.0%)
Work experience (>15 years)	8(100.0%)

S2.3 Coordination of expert opinions

The coefficient of variation for the importance ratings of each indicator in the two rounds of surveys is 0-0.25, indicating a low degree of opinion dispersion. The Kendall coordination coefficients for the two rounds of investigation are shown in Table 3. The coordination coefficient of the first round of investigation was fair, but the chi square test showed $p<0.05$, indicating that the results are acceptable. The coordination coefficient of the second round of investigation is moderate, and the chi square test is $p<0.05$. Therefore, the expert group discussed and finalized it.

Table S3 Kendall coordination coefficient and chi square test

	First round	Second round
W	0.265	0.410
Chi square	59.46	91.803

df	7	7
p	<0.001	<0.001

S2.4 Pilot survey

To improve the wording to ensure quality of the answers, a group of 10 physicians was enrolled in a pilot study to evaluate the platform used and item comprehensibility. The final questionnaire underwent minor rephrasing and formatting revisions before use.
