# Supplementary material for: Shared Decision-Making on Life-Sustaining Treatment: A Survey of Current Barriers in Practice Among Clinicians Across China
Source: Healthcare (Basel). 2025 Mar 3;13(5):547. doi: 10.3390/healthcare13050547 (PMC11898668; doi:10.3390/healthcare13050547)
Supplement: Supplementary file 1 [file healthcare-13-00547-s001.zip › supplementary file S2.pdf]

Dear doctor

We would like to invite you to complete a questionnaire survey on communication regarding the condition of critically ill patients. Please read the following information carefully. If you need further information, please ask the researchers.

Research purpose

The purpose of this study is to understand the current status of doctor-patient/family communication in critically ill patients, identify potential issues, and promote the optimization of the communication process. At the same time, we will organize corresponding suggestions based on your feedback, in order to improve the efficiency of communication and provide you with more decision-making assistance in the future, in order to improve the quality of medical services for critically ill patients.

Research process

This study will be conducted in the form of online surveys. In the survey, you will need to fill in some basic personal information. Then, you need to express your attitude towards some issues related to communication. The entire investigation will take approximately 20 minutes. If you are willing to, you can also freely express and add your own opinions.

Research risk

When answering the question, you need to recall the process of communication regarding recent medical encounter, but completing this survey does not pose any known risks. You can refuse to answer some or all of the questions. If you are willing to, you can terminate your questionnaire filling at any time.

The benefits of participating in surveys

This study did not directly benefit the participants. But your answer can help researchers better understand the obstacles in patient communication and improve the quality of medical services.

Confidentiality investigation

Your answer is completely anonymous and kept confidential by our research team, and it does not affect the patient's subsequent treatment decisions. Please do not disclose any identity information in the questionnaire.

Voluntary participation

You voluntarily participate in this study. You can withdraw at any time without reason.

Contact information

If you have any questions about this study, please contact Dr. Li at 15611963505.

Informed consent

I read and understand the information provided, and have the opportunity to raise questions. I voluntarily participate in the investigation and can withdraw at any time without reason.

Signature: Date: Month/Day/Year

Next, please think of the communication process of the doctor-patient/family conversation(DPC) on life-sustaining treatment(LST) you have encountered. Check the option that is closest to your own feelings. Unless otherwise specified, all are "single-choice questions".

1.What do you think is the main barriers to doctor-patient conversations

☐ Patient/family member expectations of disease prognosis

☐ Lack of patient/family member medical knowledge

☐ Patient/family members negative emotional status

☐ Neglected patient autonomy

☐ Lack of time due to physician workload

☐ Lack of communication skills of health care professionals

☐ Insufficient physician understanding of the disease and ability to cope with difficult situations

☐ other

2. Which process do you take during DPC of LST

□shared decision-making process

□patient-centered

□follow family choice

□paternalism mode

3. Do you think critically ill patients has decisional capacity?

□full capable

□mostly capable

□partly capable

□not capable

4. Who you make informed consent of LST to?

□patients’ family

□patient

5. Do you ask about patients’ advanced directives

□always

□sometimes

□occasionally

□never

6. Do you ask patients’ families about the patients’ LST preferences

□always

□sometimes

□occasionally

□never

7. How you rate patients/surrogates’ abilities to make medical decisions

□excellent

□good

□moderate

□insufficient

□extremely insufficient

8. How about the patients/families’ understandings of the necessity and urgency of LST

□excellent

□good

□moderate

□insufficient

□extremely insufficient

9.How about the patients/families’ comprehensions of the risk and prognosis of patients receiving LST

□excellent

□good

□moderate

□insufficient

□extremely insufficient

10. What do you think of the decisions of the patient/family. Are they concordant with your professional judgment?

□usually

□sometimes

□occasionally

□rarely

11. Would the patient/family adhere to their decisions?

□usually

□sometimes

□occasionally

□never

12. What are the factors that you considered appropriate to evaluate whether patients could benefit from receiving LST included disease prognosis

□Disease prognosis

□Comorbidity and living status

□Patient age

□Patient values

□Treatment expenses

□Other

13.Do you review patients' comorbidities and daily living statuses, respectively, during discussions with patients/families

□usually

□sometimes

□occasionally

□never

14. Do you explain the main problems and general prognoses of diseases

□usually

□sometimes

□occasionally

□never

15.Do you use tools (e.g., web videos, pictures, etc.)

□usually

□sometimes

□occasionally

□never

16. Do you mention alternatives to LST, such as epinephrine and oxygen masks

□usually

□sometimes

□occasionally

□never

17. Do you mention the consequences of forgoing LST, such as possible death of the patient

□usually

□sometimes

□occasionally

□never

18. If patients/family members asked about the success rate of CPR, what would you answer?

□It is difficult to predict precisely for each individual

□Estimate the success rate according to literature evidence

19. What is the exact rate of primary success of return of spontaneous circulation (ROSC) for in-hospital cardiac arrest (IHCA)

20. What is the exact rate of bone fraction caused by chest compressions

21. When a patient is facing life-threatening needs for LST but their family members are not present/have not consented, you will

□immediately and actively take necessary measures to contact the family members and gain their consent before deciding on the next step of treatment

□follow an active code or "slow code" based on my evaluation of the prognosis and ability to contact family members during that time

22.How long do you need to finish DPC of LST

□<5 min

□5~15 min

□>15 min

23. When do you think should talk about CPR

□when any potential for its use was present

□when a patient was deteriorating

The following is some of your personal information that we will strictly keep confidential.

1. Your gender: □Female □Male

2. Your age: ____ years

3. Your length of service in healthcare facilities: ____years

4. The level of your hospital is

□Tertiary hospital

□County/regional hospital

□Community hospital

5. Your title

□Senior

□Median

□Junior

6. Your specialty

□Emergency medicine

□Internal medicine

□Surgery

□Intensive medicine

□Cardiology

□Other

7. Your working time per week

□40~50 hours

□50~60 hours

□>60 hours

8. How often do you perform CPR

□Often

□Sometimes

□Occasionally

□Never

9. How many times did you perform CPR during last month

□⩾5 times

□2~4 times

□⩽1 time

10. Have you ever received doctor-patient communication training

□Yes

□No

This survey has been completed completely. Thank you again for your participation and help!
