# Supplementary material for: Shared Decision-Making on Life-Sustaining Treatment: A Survey of Current Barriers in Practice Among Clinicians Across China
Source: Healthcare (Basel). 2025 Mar 3;13(5):547. doi: 10.3390/healthcare13050547 (PMC11898668; doi:10.3390/healthcare13050547)
Supplement: Supplementary file 1 [file healthcare-13-00547-s001.zip › Supplementary file S3.pdf]

Supplementary Table S1 Exploratory factor analysis and structural equation model

Factor	op	item	est	se	z	p
DMA	=~	X34	1.000	<0.001		
DMA	=~	X35	1.013	0.054	18.739	<0.001
DMA	=~	X36	0.918	0.051	18.116	<0.001
DMA	=~	X37	0.838	0.054	15.438	<0.001
DMA	=~	X42	0.541	0.049	10.998	<0.001
SY	=~	X54	1.000	<0.001		
SY	=~	X55	0.071	0.002	29.613	<0.001
SY	=~	X52	0.967	0.018	52.672	<0.001
LSTE	=~	X60	1.000	<0.001		
LSTE	=~	X61	0.866	0.040	21.789	<0.001
LSTE	=~	X62	0.774	0.045	17.030	<0.001
LSTE	=~	X58	1.631	0.103	15.815	<0.001
PV	=~	X10	1.000	<0.001		
PV	=~	X13	0.670	0.095	7.035	<0.001
PV	=~	X72	0.219	0.029	7.667	<0.001
PV	=~	X78	0.168	0.026	6.371	<0.001
PV	=~	X32	1.024	0.117	8.727	<0.001
DP	=~	X18	1.000	<0.001		
DP	=~	X20	0.212	0.066	3.205	0.001
DP	=~	X28	0.439	0.123	3.577	<0.001
DP	=~	X26	-0.025	0.101	-0.243	0.808
DP	=~	X38	0.604	0.098	6.179	<0.001
DP	=~	X40	2.070	0.274	7.564	<0.001
BDPC	=~	X16	1.000	<0.001		
BDPC	~	DMA	0.011	0.052	0.216	0.829
BDPC	~	SY	-0.008	0.003	-2.407	0.016
BDPC	~	LSTE	-0.038	0.039	-0.985	0.325

Factor	op	item	est	se	z	p
BDPC	~	PV	-0.614	0.476	-1.290	0.197
BDPC	~	DP	1.190	0.859	1.385	0.166
X34	~~	X34	0.342	0.024	14.531	<0.001
X35	~~	X35	0.335	0.024	14.257	<0.001
X36	~~	X36	0.338	0.022	15.420	<0.001
X37	~~	X37	0.532	0.030	17.822	<0.001
X42	~~	X42	0.570	0.029	19.425	<0.001
X54	~~	X54	4.670	0.885	5.276	<0.001
X55	~~	X55	0.269	0.014	19.366	<0.001
X52	~~	X52	6.215	0.856	7.258	<0.001
X60	~~	X60	0.403	0.031	12.940	<0.001
X61	~~	X61	0.178	0.020	9.049	<0.001
X62	~~	X62	0.680	0.038	18.130	<0.001
X58	~~	X58	3.738	0.201	18.591	<0.001
X10	~~	X10	0.723	0.043	16.716	<0.001
X13	~~	X13	0.703	0.038	18.752	<0.001
X72	~~	X72	0.056	0.003	18.148	<0.001
X78	~~	X78	0.060	0.003	19.198	<0.001
X32	~~	X32	0.670	0.041	16.205	<0.001
X18	~~	X18	0.470	0.026	18.427	<0.001
X20	~~	X20	0.160	0.008	20.171	<0.001
X28	~~	X28	0.530	0.026	20.098	<0.001
X26	~~	X26	0.431	0.021	20.419	<0.001
X38	~~	X38	0.204	0.011	18.759	<0.001
X40	~~	X40	0.680	0.050	13.476	<0.001
X16	~~	X16	<0.001	<0.001		
DMA	~~	DMA	0.425	0.037	11.340	<0.001
SY	~~	SY	66.560	3.584	18.571	<0.001

Factor	op	item	est	se	z	p
LSTE	~~	LSTE	0.684	0.055	12.435	<0.001
PV	~~	PV	0.232	0.039	5.883	<0.001
DP	~~	DP	0.078	0.018	4.428	<0.001
BDPC	~~	BDPC	0.301	0.026	11.715	<0.001
DMA	~~	SY	0.441	0.209	2.109	0.035
DMA	~~	LSTE	0.004	0.022	0.198	0.843
DMA	~~	PV	0.118	0.019	6.137	<0.001
DMA	~~	DP	0.074	0.013	5.597	<0.001
SY	~~	LSTE	-1.158	0.264	-4.390	<0.001
SY	~~	PV	-0.225	0.191	-1.180	0.238
SY	~~	DP	-0.060	0.119	-0.503	0.615
LSTE	~~	PV	0.035	0.021	1.706	0.088
LSTE	~~	DP	0.034	0.013	2.540	0.011
PV	~~	DP	0.120	0.018	6.691	<0.001

Abbreviations: BDPC: barriers of doctor-patient conversation on LST; **SY**: physician service year; X5-4: year of service; X5-5: title; X5-2: physician age; **LSTE**: life-sustaining treatment experience; X6-0: code leader; X6-1: familiar with LST; X6-2L: frequency of code activation in past month; X5-8: field of expertise; **DP**: disclosure pattern; X1-6: conversation based on physician habit or patient's condition;X1-8:review comorbidity and daily living status; X2-0: Explain the main issue and general prognosis; X2-6: Mention the consequences of forgoing LST ; X2-8: Mention alternatives to LST; X3-8: explain actively; X4-0: use tool; **PV**: patient value expression; X1-0: acquirement of advance directive; X1-3: patient decisional capacity; X3-2:ask for patient value;X7-2: consent to patient; X7-8: respect patient value; **DMA**: decision-making ability: X3-4: Understanding of the necessity and urgency of LST; X3-5: Comprehension of risk and prognosis of patients receive LST;X3-6: patient/surrogate decision-making ability; X3-7:stick to decision; X4-2:patient/surrogate decision concordance with physician.
